# Tsg101 Is Involved in the Sorting and Re-Distribution of Glucose Transporter-4 to the Sarcolemma Membrane of Cardiac Myocytes

**DOI:** 10.3390/cells9091936

**Published:** 2020-08-21

**Authors:** Kobina Essandoh, Shan Deng, Xiaohong Wang, Yutian Li, Qianqian Li, Xingjiang Mu, Tianqing Peng, Guo-Chang Fan

**Affiliations:** 1 Department of Pharmacology and Systems Physiology, University of Cincinnati College of Medicine, Cincinnati, OH 45267, USA; essandkq@mail.uc.edu (K.E.); dengshan1020@hust.edu.cn (S.D.); wang2xn@ucmail.uc.edu (X.W.); li3yt@ucmail.uc.edu (Y.L.); liq9@mail.uc.edu (Q.L.); muxingjiang@163.com (X.M.); 2 Department of Cardiology, Union Hospital, Tongji Medical College, Huazhong University of Science and Technology, Wuhan 430022, China; 3 Division of Pharmaceutical Sciences, James L. Winkle College of Pharmacy, University of Cincinnati, Cincinnati, OH 45267, USA; 4 Critical Illness Research, Lawson Health Research Institute, London, ON N6A 4G5, Canada; tpeng2@uwo.ca

**Keywords:** Tsg101, ischemia, hypoxia, Glut-4, myocardial infarction, endosomal recycling

## Abstract

Cardiac cells can adapt to pathological stress-induced energy crisis by shifting from fatty acid oxidation to glycolysis. However, the use of glucose-insulin-potassium (GIK) solution in patients undergoing cardiac surgery does not alleviate ischemia/reperfusion (I/R)-induced energy shortage. This indicates that insulin-mediated translocation of glucose transporter-4 (Glut-4) is impaired in ischemic hearts. Indeed, cardiac myocytes contain two intracellular populations of Glut-4: an insulin-dependent non-endosomal pool (also referred to as Glut-4 storage vesicles, GSVs) and an insulin-independent endosomal pool. Tumor susceptibility gene 101 (Tsg101) has been implicated in the endosomal recycling of membrane proteins. In this study, we aimed to examine whether Tsg101 regulated the sorting and re-distribution of Glut-4 to the sarcolemma membrane of cardiomyocytes under basal and ischemic conditions, using gain- and loss-of-function approaches. Forced overexpression of Tsg101 in mouse hearts and isolated cardiomyocytes could promote Glut-4 re-distribution to the sarcolemma, leading to enhanced glucose entry and adenosine triphosphate (ATP) generation in I/R hearts which in turn, attenuation of I/R-induced cardiac dysfunction. Conversely, knockdown of Tsg101 in cardiac myocytes exhibited opposite effects. Mechanistically, we identified that Tsg101 could interact and co-localize with Glut-4 in the sarcolemma membrane of cardiomyocytes. Our findings define Tsg101 as a novel regulator of cardiac Glut-4 trafficking, which may provide a new therapeutic strategy for the treatment of ischemic heart disease.

## 1. Introduction

The heart can adapt a variety of substrates in order to produce sufficient amounts of ATP as energy to maintain effective contractile function [1,2,3,4]. Under normal physiological conditions, the majority of cardiac energy is derived from fatty acid oxidation, but myocardial cells can rapidly change their energy source to glycolysis in response to insulin, ischemia, and increased workload [5,6]. Switching substrates from fatty acid to glucose is an important compensatory mechanism used by cardiomyocytes, especially in surgically induced ischemic/reperfused (I/R) hearts [1,2,3,4]. Thus, strategies that enhance glucose uptake represent a promising approach to treat ischemic heart disease. Theoretically, insulin, well-known to stimulate glucose entry and utilization [7,8], is expected to provide enhanced protection against I/R-induced injury. However, recent clinical and lab studies indicate that ischemia induces a reduction of intracellular pH, which in turn inhibits cardiac insulin signaling [9,10,11]. Accordingly, multiple recent clinical trials testing the effects of glucose-insulin-potassium (GIK solution) on patients undergoing heart surgery have not yielded beneficial outcomes [12,13,14]. These studies suggest that insulin resistance in surgically induced I/R hearts remains a major hurdle in treating such patients.

Given that glucose cannot permeate the hydrophobic lipid bilayer of the plasma membrane, glucose is taken up into the cell by various glucose transporters [4]. Under basal conditions, a large amount of glucose transporter-4 (Glut-4), the major isoform of glucose transporters in adult hearts, is kinetically sequestered in GSVs (Glut-4 storage vesicles, clustered in the vicinity of endocytic re-cycling compartment throughout the cytosol), and only a small fraction is distributed to endosomes, the trans-Golgi network (TGN), and the plasma membrane [4,15,16]. At present, it is widely recognized that translocation of Glut4 from intracellular compartments (i.e., GSVs and endosomes) to the plasma membrane can be regulated by insulin and its downstream phosphoinositide 3-kinase (PI3K)/Akt pathway [7,8]. Nonetheless, recent studies have implicated that compensatory elevation of plasma Glut-4 in the ischemic heart largely originate from insulin-resistant endosomes rather than insulin-sensitive GSVs [10,11,15]. Moreover, Tian et al. demonstrated that mice lacking cardiac Glut-4 develop moderate hypertrophy and fail to protect from ischemia-induced ATP shortage and cardiac dysfunction [17]. Thus, it would be an advantage to promote insulin-independent endosomal recycling of Glut-4 for treating cardiac ischemia, in terms of I/R-triggered insulin resistance.

Tumor susceptibility gene 101 (Tsg101) was initially linked to tumorigenesis in various animal and human cancer models [18,19]. Subsequently, several studies indicated that Tsg101 functions as an essential component of the endosomal sorting complexes required for transport (ESCRT) machinery [20,21], and it is actively involved in the endosomal recycling of receptors to the plasma membrane and trans-Golgi network [22,23]. In particular, Tsg101 has been demonstrated to recruit and transfer epidermal growth factor receptor (EGFR) from recycling endosomes to the plasma membrane in tumor cells [22,23]. Most recently, our lab observed that Tsg101 promoted the endosomal-mediated transport of the insulin-like growth factor-1 receptor (IGF-1R) to the plasma membrane (sarcolemma) of cardiomyocytes, leading to physiological cardiac hypertrophy [24]. Furthermore, we observed that overexpression of Tsg101 in the heart protected against I/R-induced cardiac injury [25]. Hence, these novel findings prompted us to test whether Tsg101 could positively regulate sarcolemma Glut-4 levels in I/R hearts through endosomal-mediated recycling.

In this study, we first determined the sarcolemma expression levels of Glut-4 and Tsg101 in ischemic hearts. Next, mice with cardiac-specific overexpression of Tsg101 and inducible heart-specific knockdown of Tsg101 were utilized to measure subcellular distribution of Glut-4, cardiac glucose uptake, ATP generation and contractile function upon ex vivo I/R. On the other hand, neonatal cardiomyocytes were isolated to investigate the effects of Tsg101-elevation and -reduction on glucose entry and cell survival upon hypoxic conditions. Finally, we identified that Tsg101 could interact with Glut-4, which promoted its sorting and re-distribution to the sarcolemma membrane of cardiomyocytes.

## 2. Materials and Methods

### 2.1. Animals

The mouse model with heart-specific overexpression of Tsg101 is of FVB/N background and was generated by the Transgenic Animal and Genome Editing Core at Cincinnati Children’s Hospital Center (Cincinnati, OH, USA) as described previously [26]. Inducible cardiac-specific Tsg101-knockdown (KD) mouse model was generated by mating female mice harboring the Tsg101 floxed allele (Tsg101fl/fl,129/SvJ background) with male mice expressing the inducible Cre recombinase transgene under the control of the αMHC promoter (αMHC-Mer-Cre-Mer, C57BL/6 background). Male hetero-zygotes (MerCreMer-Tsg101fl/+) offspring from this cross were injected with tamoxifen (Sigma, St. Louis, MO, USA, 30 μg/g) at eight-weeks-old for three consecutive days to obtain knockdown of Tsg101 in the heart [26]. Previous work had indicated that complete knockout of Tsg101 in mice was lethal [27]. Mice expressing an inducible Cre recombinase transgene driven by the αMHC promoter αMHC-MerCreMer were purchased from the Jackson Laboratory (Stock No: 005657). Control (CTRL) mice were Tsg101fl/+ male mice of the same genetic background as Tsg101-KD mice. All mice used in this study were maintained and bred in the Division of Laboratory Animal Resources at the University of Cincinnati Medical Center. Animal experiments conformed to the Guidelines for the Care and Use of Laboratory Animals prepared by the National Academy of Sciences, published by the National Institutes of Health, and approved by the University of Cincinnati Animal Care and Use Committee.

### 2.2. Ex Vivo Cardiac Ischemia/Reperfusion

The Langendorff perfusion apparatus was used for the ex vivo model of ischemia/reperfusion in mouse hearts as described previously with few modifications [25]. In brief, mice were anesthetized, and hearts were excised and promptly cannulated and mounted to the Langendorff retrograde heart perfusion system. The phosphate-free Krebs–Henseleit buffer (118 mM NaCl, 4.7 mM KCl, 1.2 mM MgSO_4_, 1.2 mM KH_2_PO_4_, 2.5 mM CaCl_2_, 25 mM NaHCO_3_, 0.003 mM Na_2_EDTA, 11 mM Glucose pH 7.4), equilibrated at 37 °C with 5% CO_2_/95% O_2_, was used to perfuse hearts for 10 min. Afterward, mouse hearts were subjected to no-flow ischemia for 45 min. A set of mouse hearts used to determine cardiac contractile function, glucose uptake and energy utilization underwent 30-min reperfusion after ischemia. During reperfusion, cardiac contractile parameters left ventricular diastolic pressure (LVDP), rate of contraction (dP/dt) and rate of relaxation (dP/dt) were recorded with a DigiMed Heart Performance Analyzer (Micro-Med., Waukesha, WI, USA).

### 2.3. Isolation of Neonatal Rat Cardiomyocytes and Transfection with Recombinant Adenoviruses

One- to three-day-old Sprague Dawley rat neonates were anesthetized on ice, and hearts were dissected under sterile conditions. Using the Worthington Neonatal Cardiomyocytes Isolation System (Worthington Biochemical Corporation, NJ, USA), we isolated neonatal rat cardiomyocytes (NRCMs), according to the manufacturer’s procedures. NRCMs were seeded in 100-mm culture dishes for the subsequent treatment and Western-blotting analysis, in six-well plates for glucose uptake and ATP content assays and in 96-well plates for cell viability assay. After 48 h culture, NRCMs were transfected with adenoviruses expressing green fluorescent protein (GFP)-fused Tsg101 (Ad.Tsg101) and adenoviruses expressing shRNA targeted to Tsg101 (Ad.shTsg101) for 48 h. NRCMs transfected with adenoviruses expressing GFP (Ad.GFP) and adenoviruses expressing shRNA targeted to GFP (Ad.shGFP) were used as controls. Subsequently, NRCMs were treated with 200 μM H_2_O_2_ to induce hypoxia or equivalent volume of PBS as control conditions.

### 2.4. Measurement of Glucose Uptake

Glucose uptake in ex vivo I/R hearts was measured using the 2-DG Uptake Measurement kit (Cosmo Bio Co. Ltd., Tokyo, Japan) following the manufacturer’s instructions. After 10 min equili-bration period with phosphate-free Krebs–Henseleit buffer, the Langerdoff hearts were perfused with Krebs Ringer Phosphate Hepes (KRPH) buffer (1.2 mM KH_2_PO_4_, 1.2 mM MgSO_4_, 1.3 mM CaCl_2_, 118 mM NaCl, 5 mMKCl, 30 mM Hepes, pH 7.5) containing 5 mM of the glucose analog 2-deoxy-D-glucose (2-DG) for 30 min, before the I/R protocol. Afterward, hearts were homogenized in 10 mM Tris-HCL (pH 8.0), heated at 80 °C for 15 min, and centrifuged at 15,000× *g* for 15 min at 4 °C. Then, supernatants were diluted five times and subjected to the kit to measure intracellular 2-DG as a rate of glucose transport into heart tissue. For the in vitro glucose uptake assay, NRCMs were cultured to about 80% confluence and transfected with corresponding recombinant adenoviruses in six-well plates. Cells were washed with PBS twice and cultured in KRPH/2% bovine serum albumin (BSA) solution for 40 min. Cells were then stimulated with 200 μM H_2_O_2_ for 20 min and treated with 2-DG for an additional 20 min. Next, cells were extracted, and subjected to measure 2-DG6P levels as a rate of glucose transport into cardiomyocytes, using a Glucose Uptake Assay Kit (ab136955, Abcam, Cambridge, MA, USA).

### 2.5. Measurement of ATP Content and Cell Viability

The ATP content in mouse hearts and NRCMs were determined using the ATP Assay Kit (ab83355, Abcam, Cambridge, UK). Briefly, heart tissue and cardiomyocyte samples were homogenized in ATP Assay buffer and centrifuged at 13,000× *g* for 5 min at 4 °C. The resulting supernatant was processed with Deproteinizing Sample Preparation Kit-TCA (ab204708, Abcam) to remove enzymes that may disrupt ATP quantification. ATP content in the processed samples were then measured at OD = 570 nm with a microplate reader. Cardiomyocyte survival was assessed using the MTS assay kit (ab197010, Abcam) according to the manufacturer’s instructions. Briefly, NRCMs were seeded and transfected with corresponding recombinant adenoviruses in 96-well plates. Cells were treated with 200 μM H_2_O_2_ or PBS as control for 4 h. The MTS reagent was added to the cells and incubated for 2 h at 37 °C. Absorbance was read at OD = 490 nm as relative cell viability.

### 2.6. Western Blotting and Co-Immunoprecipitation (Co-IP) Analysis

Total proteins were extracted from hearts using the NP40 lysis buffer (completeTM, Mini) supplemented with protease inhibitor cocktail (Roche, Basel, Switzerland), according to manufac-turer’s instructions. Plasma membrane (PM) and PM/nuclei-depleted intracellular protein were isolated/separated using the Minute Plasma Membrane Protein Isolation Kit (Invent Biotechnologies, Plymouth, MN, USA) by following the manufacturer’s protocol. Protein concentrations of total, PM/nuclei-depleted intra-cellular protein and plasma membrane protein were determined by Protein Assay Reagent (Bio-Rad, Hercules, CA, USA). Samples (10–100 µg) were loaded to SDS-PAGE as described in detail elsewhere [24]. The dilutions and sources of the primary antibodies used in this study are as follows: Tsg101 (Santa Cruz, Dallas, TX, USA, 1:1000); Glut-4 (Abcam, 1:1000); Na/K-ATPase (Cell Signaling, Danvers, MA, USA, 1:1000); Glyceraldehyde-3-phosphate dehydrogenase (GAPDH, Cell Signaling, 1:500). Western-blot bands were quantified by Multi-Image II (Alpha Innotech, San Leandro, CA, USA). The relative levels of total and PM/nuclei-depleted intra-cellular protein were normalized to GAPDH while the relative plasma membrane protein levels were normalized to sodium–potassium adenosine triphosphatase (Na/K-ATPase). As for co-IP analysis, 500 mg of mouse heart homogenates were solubilized in 1 mL NP40 lysis buffer (with protease inhibitor cocktail), and pre-cleared in 50 μL of protein A/G-agarose beads (Cell Signaling) for 1 h at 4 °C on a rotary wheel. The mixture was then incubated with 1 μg of corresponding primary antibodies at 4 °C overnight on a rotary wheel. Immunoprecipitates were collected and washed 5 times with NP40 lysis buffer. Immunoprecipitates were resolved in 2× Laemmli sample buffer and boiled at 95 °C for 5 min. Eluted proteins from the antibody-beads conjugate were subjected to SDS-PAGE.

### 2.7. Immunofluorescence Staining Analysis

Pre-ischemic (Pre-I) and post-ischemic (Post-I) hearts were fixed overnight in 10% neutral-buffered formalin for 48 h and paraffin-embedded at a thickness of 5 μm. To prepare for immunofluorescence staining, heart sections were deparaffinized in three washes of xylene and dehydrated in two washes of 100% ethanol and two washes of 90% ethanol. Sections were then incubated with primary antibodies for Tsg101 and Glut-4 overnight at 4 °C, followed by incubation with their respective fluorescence conjugated secondary antibodies for 1 h at room temperature. The sections were washed in PBS and mounted with ProLong™ Gold Antifade Mountant with DAPI (Invitrogen, Carlsbad, CA, USA). Fluorescent images were taken with confocal microscope LSM 710 (Carl Zeiss Micro-imaging, Jena, Germany).

### 2.8. Statistical Analysis

Data were expressed as means ± SD. Significance was determined by Student’s *t* test and one- or two-way ANOVA to determine differences within groups where appropriate. A value of *p* < 0.05 was considered statistically significant.

## 3. Results

### 3.1. Tsg101 and Sarcolemma Glut-4 Levels Are Upregulated in Ischemic Hearts

**Figure 1:**
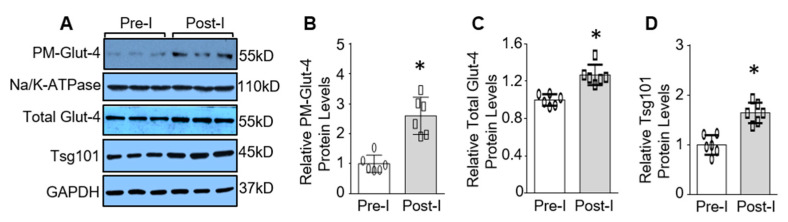
Tsg101 and sarcolemma glucose transporter-4 (Glut-4) levels are upregulated in ischemic hearts. (**A**) Western blots and quantification analysis showing the expression levels of plasma membrane (PM) (**B**) and total Glut-4 (**C**) as well as Tsg101 (**D**) in ex vivo mouse hearts subjected to 45-min ischemia. Na/K-ATPase was used as loading controls for membrane protein and GAPDH was used as loading controls for total protein. *, *p* < 0.05; *n* = 6.

Cardiac tissue switches from fatty acid oxidation to anaerobic glycolysis during ischemic events [5,6]. Accordingly, enhanced glucose uptake is essential for the cardiac contractile function recovery and cardiomyocyte cell survival during ischemia/reperfusion [17]. Given that Glut-4 is the predominant glucose transporter in the adult myocardium [16,28], we then first measured the cell membrane levels of Glut-4 in mouse hearts upon 45 min ex vivo no-flow ischemia, using the Langendorff retrograde perfusion system. Consistent with previous publications [16,28], ischemic injury resulted in increased levels of Glut-4 on the plasma membrane 2.6-fold compared to pre-ischemic heart tissue (Figure 1A,B). Interestingly, we observed that total Glut-4 protein levels were also significantly increased by 1.3-fold in the post-ischemic hearts, compared to controls (Figure 1A,C). Considering that recent studies have implicated Tsg101 as a critical mediator for the endosome-mediated trafficking of proteins to the plasma membrane [22,23,24], we next sought to determine whether Tsg101 protein levels were dysregulated in mouse hearts upon ischemic insult. Western blotting analysis showed that total protein levels of Tsg101 were elevated 1.6-fold in ischemic hearts in comparison with pre-ischemic controls (Figure 1A,D). These results suggest that Tsg101 may facilitate Glut-4 translocation to the sarcolemma in ischemic hearts.

### 3.2. Cardiac-Specific Overexpression of Tsg101 Elevates Sarcolemma Levels of Glut-4 in Hearts

**Figure 2:**
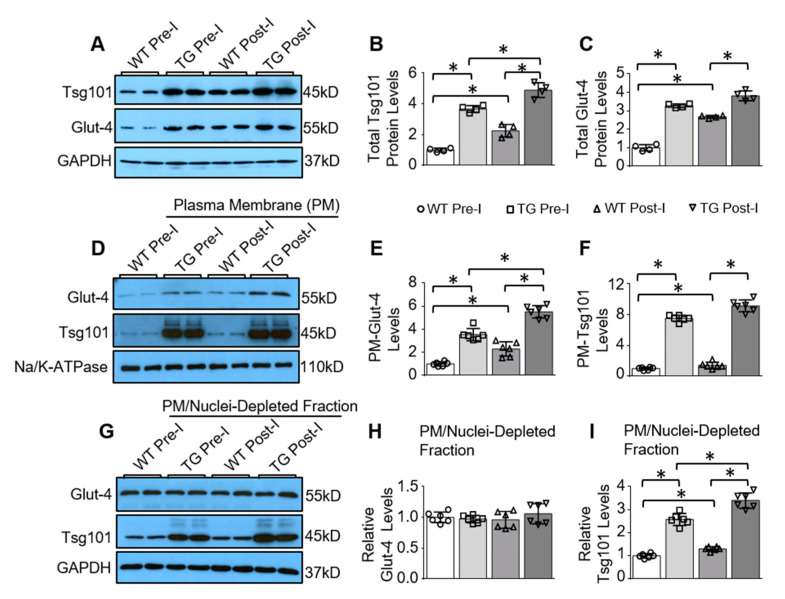
Cardiac-specific overexpression of Tsg101 in mice elevates sarcolemma levels of Glut-4 in ischemic hearts. (**A**) Representative Western blots and (**B**,**C**) quantification analysis showing total Tsg101 and Glut-4 levels in WT and Tsg101-TG hearts under pre-ischemia (Pre-I) and post-ischemia (Post-I) conditions. (**D**) Representative Western blots and (**E**,**F**) quantification analysis showing plasma membrane (PM) levels of Glut-4 (**E**) and Tsg101 (**F**) in WT and Tsg101-TG hearts under pre- and post-ischemia conditions. (**G**) Representative Western blots and (**H**,**I**) quantification analysis showing Glut-4 and Tsg101 levels in the PM/nuclei-depleted intracellular fraction isolated from WT and Tsg101-TG hearts under pre- and post-ischemia conditions. Na/K-ATPase was used as loading control for PM protein. GAPDH is used as loading controls for total and intracellular protein *, *p* < 0.05; *n* = 4–6.

To investigate the possible role of Tsg101 in Glut-4 translocation during ischemia, we generated a mouse model with heart-specific overexpression of Tsg101. Such transgenic (TG) mice have been phenotypically characterized in our previous publications [24,25,26] and validated here that Tsg101 was overexpressed by ~4-fold in TG hearts, compared to WT-hearts (Figure 2A,B). Interestingly, total levels of Glut-4 were also significantly higher in TG hearts than WT hearts (Figure 2A,C). To determine the subcellular levels of Glut-4 in WT and TG hearts upon ischemic injury, we isolated plasma membrane (PM) and PM/nuclei-depleted intracellular fraction from these pre- and post-ischemic hearts, using the Minute Plasma Membrane Protein Isolation Kit. Please note that PM/nuclei-depleted intracellular fraction may include GSVs, endosomes and other intracellular compartment contents. Western-blotting analysis results showed that plasma membrane levels of Glut-4 were remarkably increased 3.5-fold in TG hearts compared with WT controls under basal conditions (Figure 2D,E). As expected, ischemic injury increased membrane levels of Glut-4 in WT hearts; however, such increases were more pronounced in TG hearts (Figure 2D,E). In contrast, Glut-4 levels in the PM/nuclei-depleted intracellular fraction were unchanged in both WT and TG hearts at post-ischemia, relative to pre-ischemia conditions (Figure 2G,H). Furthermore, we found that both PM/nuclei-depleted intracellular fraction and sarcolemma Tsg101 levels were significantly augmented in both WT and TG hearts upon ischemia, compared to pre-ischemia conditions (Figure 2D,F,G,I). Collectively, these data suggest that overexpression of Tsg101 may enhance Glut-4 re-distribution to the cardiomyocyte surface under basal and ischemic conditions.

### 3.3. Overexpression of Tsg101 Enhances Glucose Uptake, ATP Generation, and Functional Recovery in Ischemic Hearts

**Figure 3:**
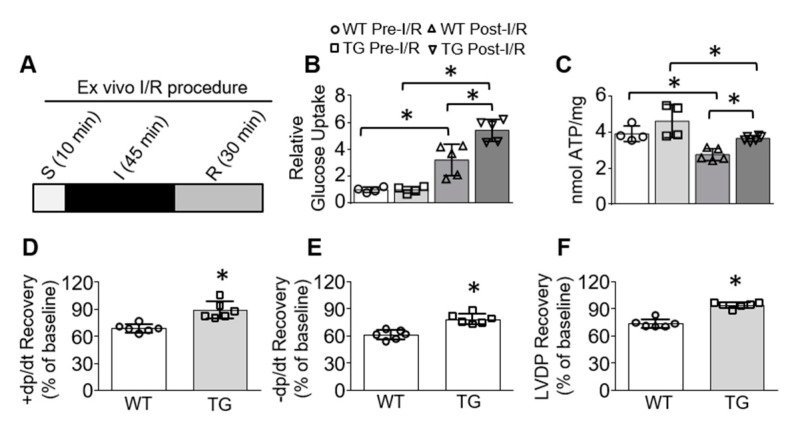
Overexpression of Tsg101 in mice improves glucose uptake, ATP content and functional recovery in ischemic hearts. (**A**) Scheme depicting experimental design for ex vivo I/R protocol in TG-hearts. (**B**) 2-deoxy-D-glucose (2-DG) uptake was measured in WT and Tsg101-TG hearts subjected to ex vivo I/R. *, *p* < 0.05; *n* = 4–5. (**C**) ATP content in WT and Tsg101-TG hearts subjected to ex vivo I/R. *, *p* < 0.05; *n* = 4–5. Recovery of (**D**) contraction (+dp/dt), (**E**) relaxation (–dp/dt) rates and (**F**) left ventricular diastolic pressure (LVDP) as a percentage of baseline in WT and Tsg101-TG hearts subjected to ex vivo I/R. *, *p* < 0.05; *n* = 6.

Rapid reperfusion of ischemic hearts could restore blood flow and reoxygenate the damaged cardiac tissue [29,30]. However, such reperfusion is riddled with overactive reactive oxygen species (ROS) generation, which further disrupts ATP production, leading to cardiac energy crisis [29,30]. Given that enhanced sarcolemma levels of Glut-4 is crucial for cardiac energy recovery from ischemic injury [17], we then asked whether increased membrane Glut-4 in TG hearts altered glucose uptake and ATP generation during reperfusion. To this end, we subjected mouse hearts to ex vivo 45-min ischemia, followed by reperfusion for 30 min (Figure 3A). By utilizing the perfusion of glucose analog, 2-deoxy-D-glucose (2-DG), in the ex vivo Langendorff system, we observed that there were no significant differences in glucose uptake in WT and TG hearts under baseline condition (Figure 3B). However, under I/R conditions, TG hearts exhibited significantly higher levels of 2-DG when compared to WT hearts (Figure 3B). Accordingly, WT hearts showed lower levels of ATP content (2.76 ± 0.33 nmol/mg tissue) compared to TG hearts (3.66 ± 0.12 nmol/mg tissue, *p* < 0.05) after reperfusion, although both sets of groups had similar ATP content under pre-I/R conditions (Figure 3C). Please note here in this study that heart samples used for measuring 2-DG entry were not utilized for determining ATP contents, as 2-DG perfusion at 5mM is poisonous for glycolysis and would inhibit ATP generation. We next recorded cardiac contractile function during reperfusion and observed that TG hearts displayed better functional recovery than WT hearts after I/R, as measured by the percent recovery of contraction rates (+dP/dt), relaxation rates (−dP/dt,) and left ventricular developed pressure over baseline (Figure 3D–F). These data indicate that overexpression of Tsg101 could protect mice from I/R-induced cardiac energy shortage and dysfunction through augmented glucose uptake and ATP production.

### 3.4. Elevation of Tsg101 in Cardiomyocytes Improves Glucose Uptake and Cardiomyocyte Survival upon Hypoxic Conditions

**Figure 4:**
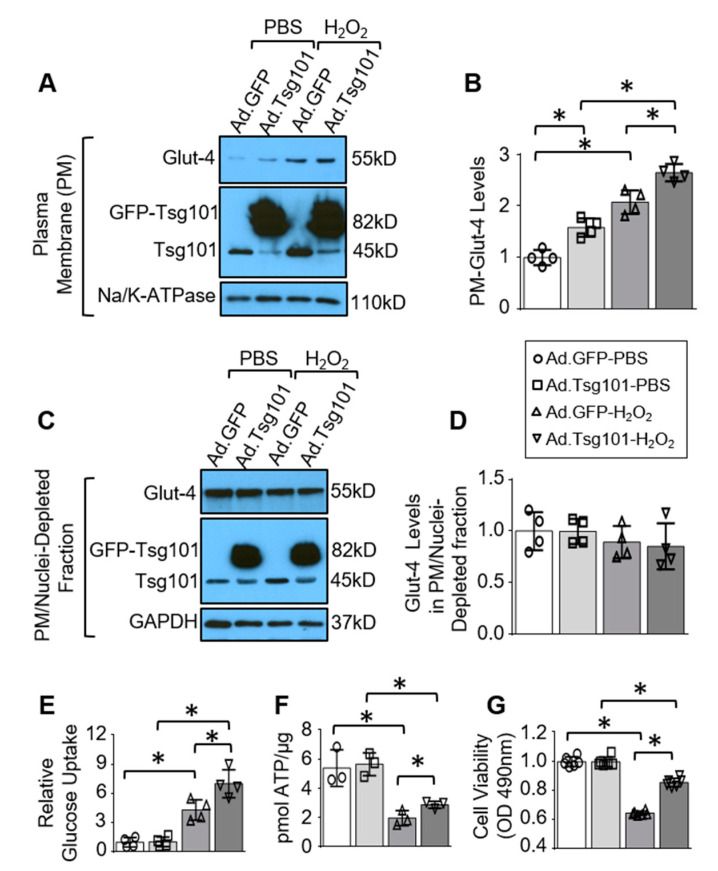
Overexpression of Tsg101 in neonatal cardiomyocytes improves glucose uptake and cell survival upon hypoxic conditions. (**A**) Western blots and (**B**) quantification analysis showing plasma membrane (PM) levels of Glut-4 and Tsg101 in neonatal rat cardiomyocytes (NRCMs) transfected with Ad.Tsg101 or Ad.GFP, then treated with H_2_O_2_ (200 μM, 2 h) or PBS. Na/K-ATPase was used as loading control for PM protein. *, *p* < 0.05; *n* = 4. (**C**) Western blots and (**D**) quantification analysis showing Glut-4 and Tsg101 levels in the PM/nuclei-depleted intracellular fraction isolated from NRCMs transfected with Ad.Tsg101 or Ad.GFP, followed by treatment with H_2_O_2_ (200 μM, 2 h) or PBS. GAPDH is used as loading controls. *, *p* < 0.05; *n* = 4. (**E**) 2-DG uptake was measured in NRCMs transfected with Ad.Tsg101 or Ad.GFP, then treated with H_2_O_2_ (200 μM) or PBS. *n* = 4. *, *p* < 0.05. (**F**) ATP content in NRCMs transfected with Ad.Tsg101 or Ad.GFP, then treated with H_2_O_2_ (200 μM) or PBS. *, *p* < 0.05; *n* = 3. (**G**) MTS analysis of cell survival in NRCMs transfected with Ad.Tsg101 or Ad.GFP, then treated with H_2_O_2_ (200 μM, 4 h) or PBS. *, *p* < 0.05; *n* = 6.

To further determine the effects of Tsg101on Glut-4 translocation in cardiac myocytes, we isolated neonatal rat cardiomyocytes (NRCMs) and transfected with adenoviruses expressing Tsg101 or control GFP. Subsequently, these cells were treated with H_2_O_2_ for 2 h to mimic the hypoxic and low-glucose conditions observed in ischemic hearts [31]. Immunoblot analysis revealed that sarcolemma levels of Glut-4 were significantly higher in Tsg101-overexpressing myocytes in normoxic conditions in relation to control cells (Figure 4A,B). Under hypoxic conditions, membrane levels of Glut-4 were greatly elevated in both Ad.GFP- and Ad.Tsg101-transfected cardiomyocytes, compared to their respective PBS-treated controls (Figure 4A/B). However, H_2_O_2_-treated Ad.Tsg101-cells exhibited significantly higher levels of membrane Glut-4 than H_2_O_2_-treated Ad.GFP-myocytes (Figure 4A,B). Similar to ex vivo intact hearts, Glut-4 levels in the PM/nuclei-depleted intracellular fraction showed no significant differences between Ad.GFP- and Ad.Tsg101-cardiomyocytes under both normoxic and hypoxic conditions (Figure 4C,D). We next evaluated whether elevated membrane levels of Glut-4 in Tsg101-overexpressing cells impacted glucose utilization in hypoxic conditions. Our analysis showed that glucose entry was enhanced in Ad.Tsg101-myocytes in relation to Ad.GFP-cells upon hypoxic conditions, while there were no changes in 2-DG levels between both groups in normal conditions (Figure 4E). Moreover, Ad.GFP- and Ad.Tsg101-transfected myocytes showed similar ATP content in PBS-treated conditions, but H_2_O_2_-induced reduction of ATP content was more drastic in Ad.GFP-cells than in Ad.Tsg101-cardiomyocytes (Figure 4F). Accordingly, elevation of Tsg101 in NRCMs boosted cell survival compared to control cells upon exposure to H_2_O_2_ (Figure 4G). Put together, these data indicate that forced overexpression of Tsg101 in NRCMs could alleviate hypoxic cell death through increased glucose utilization and ATP generation.

### 3.5. Knockdown of Tsg101 Diminishes Sarcolemma Levels of Glut-4 as Well as ATP Production and Function Recovery in Ischemic Hearts

**Figure 5:**
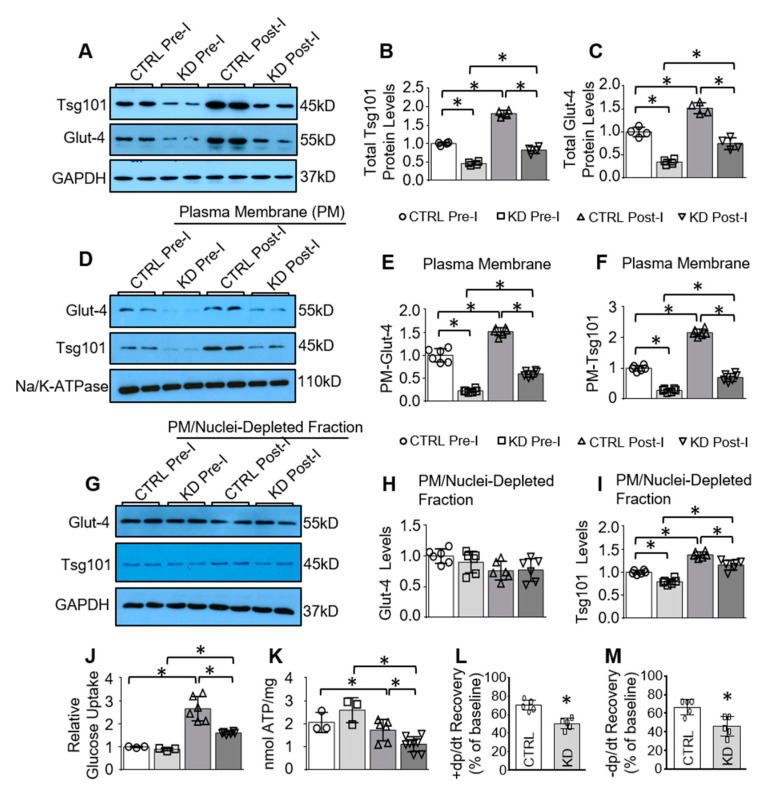
Cardiac-specific knockdown of Tsg101 diminishes sarcolemma levels of Glut-4, glucose uptake, ATP production and function recovery in ischemic hearts. (**A**) Representative Western blots and (**B**,**C**) quantification analysis showing total protein levels of Tsg101 and Glut-4 in CTRL and Tsg101-KD hearts upon pre- and post-ischemia conditions. (**D**) Representative Western blots and (**E**,**F**) quantification analysis showing plasma membrane (PM) levels of Glut-4 and Tsg101 in CTRL and Tsg101-KD hearts upon pre- and post-ischemia conditions. (**G**) Representative Western blots and (**H**,**I**) quantification analysis showing Glut-4 and Tsg101 levels in the PM/nuclei-depleted intracellular fraction isolated from CTRL and Tsg101-KD hearts upon pre- and post-ischemia conditions. Na/K-ATPase was used as loading control for PM protein. GAPDH is used as loading controls for total and intracellular protein *, *p* < 0.05; *n* = 4–6. (**J**) 2-DG uptake and (**K**) ATP content were measured in CTRL and Tsg101-KD hearts upon pre- and post-ischemia conditions *, *p* < 0.05; *n* = 3–8. (**L**,**M**) Recovery of contraction (+dp/dt) and relaxation (–dp/dt) rates (**K**) as a percentage of baseline in CTRL and Tsg101-KD hearts subjected to ex vivo I/R. *, *p* < 0.05; *n* = 5.

To further examine the role of Tsg101 on the subcellular distribution of Glut-4 during cardiac ischemia, we utilized an inducible cardiac-specific Tsg101 knockdown (Tsg101-KD) mouse model. It is important to note that complete knockout of Tsg101 in the heart is lethal for mice [24]. The generation of Tsg101-KD mice has been described previously [24] and was validated in this study by Western-blot analysis showing that Tsg101 expression was reduced by about 50% in such Tsg101-KD mouse hearts (Figure 5A,B). Interestingly, knockdown of Tsg101 in mouse hearts significantly reduced total Glut-4 levels, compared to control hearts (CTRL) (Figure 5A,C). Furthermore, we isolated plasma membrane (PM) and the PM/nuclei-depleted intracellular fraction from heart homogenates of these KD mice and littermate controls at pre- and post-ischemia (ex vivo 45-min no-flow ischemia) for Western blotting. As shown in Figure 5D,E, Tsg101-KD hearts exhibited remarkably reduced plasma membrane levels of Glut-4 under basal pre-ischemic (Pre-I) conditions, compared to CTRL hearts. Although ischemic injury elicited compensatory up-regulation of sarcolemma Glut-4 levels in CTRL hearts, such increase in plasma membrane Glut-4 level was greatly attenuated in post-ischemic KD hearts (Figure 5D,E). Similar to the TG model, cardiac ischemic injury did not elicit any significant changes in intracellular Glut-4 in both CTRL and KD hearts (Figure 5G,H). On the other hand, intracellular and membrane Tsg101 were remarkably elevated in response to ischemic injury in both CTRL and KD hearts, in comparison with pre-ischemic conditions (Figure 5D,F,G,I). Collectively, these data indicate that knockdown of Tsg101 may inhibit transport of Glut-4 to the cardiomyocyte membrane.

We next sought to elucidate whether the reduction of sarcolemma Glut-4 in KD hearts affected glucose uptake, energy production, and cardiac function during I/R. As such, CTRL and KD hearts were subjected to ex vivo 45-min ischemia, followed by 30-min reperfusion to determine glucose utilization and cardiac functional recovery. Under pre-I/R conditions, there were no significant differences in glucose uptake between CTRL and KD hearts (Figure 5J). Regardless, KD hearts showed lower levels of 2-DG uptake, compared to WT hearts upon I/R injury (Figure 5J), indicating diminished glucose entry. As a consequence, KD hearts exhibited lower levels of ATP (1.12 ± 0.33 nmol/mg tissue), in comparison with CTRL hearts (1.73 ± 0.47 nmol/mg tissue), although ATP levels were similar in CTRL and KD hearts under baseline conditions (Figure 5K). Furthermore, KD hearts showed significantly depressed functional recovery compared with CTRL hearts, as evidenced by the lower percent recovery of contraction (+dP/dt) and relaxation (−dP/dt) rates in KD hearts relative to CTRL samples (Figure 5L,M). Taken together, these results demonstrate that Tsg101 deficiency impairs glucose uptake and utilization in mouse hearts, leading to cardiac dysfunction under ex vivo I/R conditions.

### 3.6. Reduction of Tsg101 in Neonatal Myocytes Impedes Glucose Uptake, ATP Production and Promotes Cell Death upon Hypoxic Conditions

**Figure 6:**
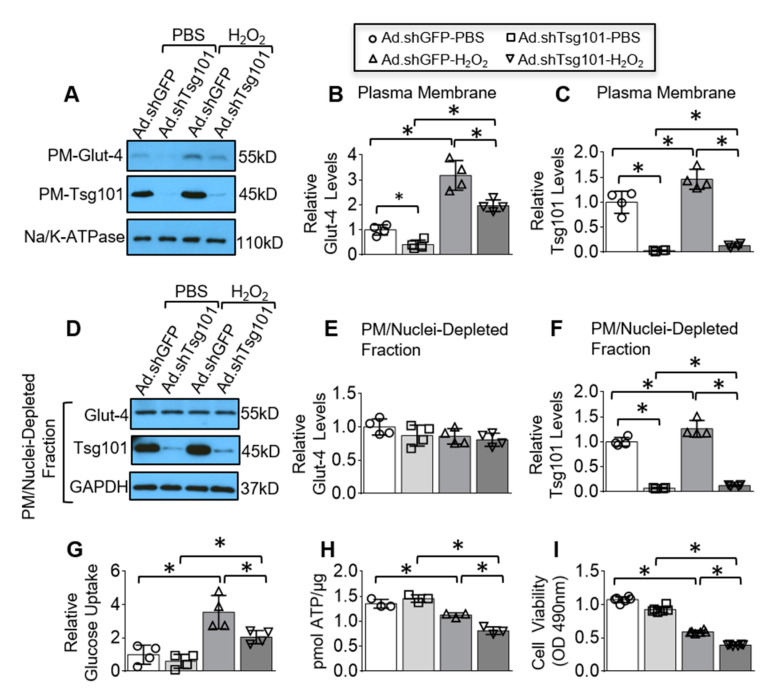
Reduction of Tsg101 in neonatal myocytes impairs glucose uptake and ATP production and promotes cell death. (**A**) Western blots and (**B**,**C**) quantification analysis showing plasma membrane (PM) levels of Glut-4 (**B**) and Tsg101(**C**) in neonatal Sprague Dawley rat cardiomyocytes (NRCMs) transfected with Ad.shTsg101 or Ad.shGFP, then treated with H_2_O_2_ (200 μM, 2 h) or PBS. Na/K-ATPase was used as loading control for PM protein. *, *p* < 0.05; *n* = 4. (**D**) Western blots and (**E**,**F**) quantification analysis showing Glut-4 and Tsg101 levels in the PM/nuclei-depleted intracellular fraction isolated from NRCMs transfected with Ad.shTsg101 or Ad.shGFP, followed by treatment with H_2_O_2_ (200 μM, 2 h) or PBS. GAPDH is used as loading controls. *, *p* < 0.05; *n* = 4. (**G**) 2-DG uptake and (**H**) ATP content as well as (**I**) MTS analysis of cell viability were measured in NRCMs transfected with Ad.shTsg101 or Ad.shGFP, followed by exposure to H_2_O_2_ (200 μM) or PBS. *, *p* < 0.05; *n* = 3–6.

To elucidate whether reduction of Tsg101 in cardiomyocytes in vitro had impacts on glucose uptake, ATP production and cell viability, we transfected NRCMs with adenoviruses that express shRNA targeted to Tsg101 (Ad.shTsg101) or Ad.shGFP, followed by treatment with H_2_O_2_ or control PBS. shRNA-mediated knockdown of Tsg101 in cardiomyocytes was confirmed in both membrane and intracellular levels, compared to shGFP cells (Figure 6A,C,D,F). Similar to ex vivo intact hearts, reduction of Tsg101 in cardiomyocytes significantly reduced the amount of plasma membrane Glut-4, compared to shGFP-cells upon normoxic conditions (Figure 6A,B). Although H_2_O_2_ elicited remarkable increase in surface levels of Glut-4 in Ad.shGFP-myocytes, the degree of such increase was greatly suppressed by knockdown of Tsg101 (Figure 6A,B). Regardless, there were no changes in intracellular levels of Glut-4 between Ad.shGFP- and Ad.shTsg101-cells after hypoxic insult, relative to PBS-treated conditions (Figure 6D,E). In line with the reduced Glut-4 level on plasma membrane in Tsg101-KD cells, glucose entry was greatly abrogated in Ad.shTsg101-myocytes after hypoxic challenge, compared to Ad.shGFP cells (Figure 6G), which led to lower levels of ATP generation (Figure 6H). Furthermore, cell viability analysis showed that knockdown of Tsg101 significantly exacerbated cardiomyocyte death in comparison with control cells after 4-h exposure to H_2_O_2_ (Figure 6I). Together, these in vitro data suggest that depletion of Tsg101 in cardiomyocytes could elicit detrimental effects on glucose uptake, ATP production, and cell death.

### 3.7. Tsg101 Interacts and Co-Localizes with Glut-4 in the Ischemic Heart

**Figure 7:**
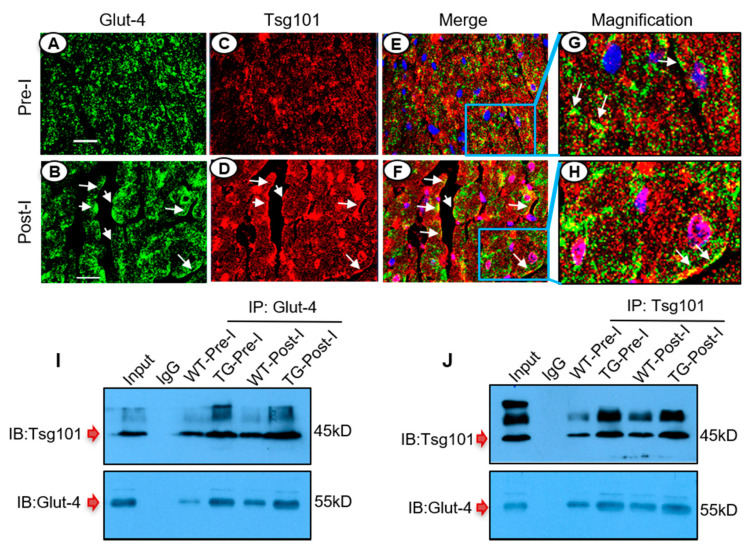
Tsg101 interacts and co-localizes with Glut-4 in the ischemic heart. Co-immunostaining of heart sections with Glut-4 antibody (green, **A**,**B**) and Tsg101 antibody (red, **C**,**D**) as well as merged (**E**,**F**). The blue squares inside (**E**,**F**) are magnified to be (**G**) and (**H**), respectively. *n* = 4 hearts, two sections per hearts. Scale bar: 100 µm. (**I**) Co-immunoprecipitation (Co-IP) using anti-Glut-4 and immunoblotting (IB) for Tsg101 in WT- and TG-hearts subjected to ex vivo 45-min ischemia. *n* = 4 for each group. (**J**) Co-IP using anti-Tsg101 and immunoblotting for Glut-4 in mouse hearts subjected to ex vivo 45-min ischemia. *n* = 4 for each group.

To dissect how Tsg101 regulates Glut-4 transport to the plasma membrane during ischemic injury, we first determined the distribution of Glut-4 and Tsg101 in mouse heart sections, collected at ex vivo pre-ischemia and post-ischemia. The immuno-staining results showed that Glut-4 was largely scattered in the cytosol of myocytes under basal conditions (Figure 7A) but was mostly accumulated at the myocyte surface upon ischemia (Figure 7B). Notably, Tsg101 was also detected as a scattered dot-pattern in the cytosol of pre-I myocytes (Figure 7C). When the green Glut-4 image (Figure 7A) was merged with the red Tsg101 image (Figure 7C), we observed that a few dots of Glut-4 co-localized with Tsg101 in the Pre-I myocytes (Figure 7E,G, yellow dots, white arrows). More interestingly, under ischemia condition, Tsg101 could be translocated to the membrane edge of myocytes (Figure 7D, white arrows), and co-localized with sarcolemma Glut-4 (Figure 7F,H, white arrows). To further examine whether Tsg101 could interact with Glut-4, we performed co-immunoprecipitation (co-IP) assays using ischemic heart homogenates. Our results showed that not only did Tsg101 bind to Glut-4, but this association was further enhanced in both WT and TG hearts after ischemic injury (Figure 7I,J). Taken together, these data suggest that Tsg101 could interact with Glut-4 and may promote its accumulation at the plasma membrane of cardiomyocytes upon ischemia.

## 4. Discussion

The present study shows a previously unrecognized role of Tsg101 in facilitating the transport of Glut-4 to the sarcolemma in ischemic hearts. Using gain- and loss-of-function approaches in ex vivo intact hearts and in vitro cardiac myocytes, we demonstrated that Tsg101 might be a critical mediator for the re-distribution of cardiac Glut-4 to the plasma membrane. Mechanistically, we identified that Tsg101 interacted with Glut-4 in the heart, which could promote recycling of Glut-4 to the plasma membrane, thereby alleviating I/R-induced ATP shortage and cardiac depression.

The major source of energy in a normal functioning heart is fatty acid oxidation. However, in ischemic stress conditions, the heart adopts glucose-mediated glycolysis which is a more efficient way of producing ATP due to lesser consumption of oxygen compared to fatty acid oxidation [32]. Sarcolemma translocation of Glut-4, which initiates glucose uptake in the adult heart, has largely been ascribed to insulin signaling and downstream activation of the PI3K/Akt pathway [7,8]. In particular, Tian et al. reported that mice with cardiac-specific knockout of Glut-4 reduced myocyte glucose utilization, glycolytic flux, ATP generation and thereby, cardiac dysfunction during ischemia/reperfusion [17]. As such, ischemic heart models are characterized by a compensatory upregulation of Glut-4 in order to effectively utilize the scarce amounts of oxygen to maintain cardiac contractile function [16,28]. Along this line, Sun et al. applied ischemia in Langendorff rat hearts which caused substantial translocation of Glut-4 from the microsomal fractions to the sarcolemma [28]. Similarly, Heather et al. showed that the sarcolemmal content of Glut-4 in male Wistar rat hearts during ischemia was increased by 90%, accompanied with an 86% increase in glycolytic rates, whereas fatty acid oxidation rates were decreased by 95% [16]. Thus, it seems logical that stimulating Glut-4 translocation to plasma membrane would be of benefit to cardiac ischemic patients. However, multiple clinical trials testing the effectiveness of administering the glucose-insulin-potassium (GIK) solution into surgical cardiac ischemic/reperfused patients has not yielded positive outcomes [9,10,11]. These observations attest to the incomplete knowledge regarding the responsiveness of ischemic hearts to insulin. Indeed, Beauloye et al. revealed that insulin signaling is impaired in ischemic hearts due to intracellular acidosis and reduction in cellular pH [9]. In addition, there is evidence to show that an initial activation of insulin/Akt pathway, one day after myocardial infarction (MI) injury in rats, was attenuated at one week after MI injury [11]. Furthermore, compensatory upregulation of glucose transporter-1 (Glut-1) in Glut-4 deficient hearts did not protect mice from ischemia-triggered cardiac dysfunction [17]. Thus, it is imperative to develop therapies that promote plasma membrane transport of Glut-4, independent of insulin, during cardiac ischemia. As a matter of fact, this study presented here for the first time showed that, in the absence of insulin, forced elevation of cardiac Tsg101 could facilitate trafficking/recycling of Glut-4 to the surface of cardiomyocytes, leading to better cardiac functional recovery after I/R injury (Figure 3D–F).

Previous work has pointed to the existence of two intracellular pools on Glut-4 stores in cardiomyocytes. One is Glut-4 storage vesicle (GSV) and has a large content of Glut-4 with minimal amounts of Glut-1 [33]. Another pool is associated with endosomes, where it has slightly less Glut-4 content but contains higher amounts of Glut-1 [33]. Interestingly, both pools can be stimulated by insulin under normal conditions [33]. Nonetheless, the endosome-associated Rab-GTPase, Rab11, has been consistently linked to trafficking of Glut-4 vesicles in cardiomyocytes [34,35,36]. Treatment of rat cardiomyocytes with insulin resulted in sarcolemma translocation of a high-density pool of Glut-4 which was localized with Rab11 and recycling endosome marker, transferrin receptor [34]. Furthermore, studies using the Rab11 dominant negative mutation (Rab11 N124I) in cardiac myoblast cell line H9c2 showed 50% reduction in plasma membrane amounts of Glut-4 after insulin stimulation [35]. The same group later demonstrated that Rab11 is GTP-loaded onto Glut-4 vesicles after insulin stimulation and Rab11-mediated Glut-4 transport is initiated by the PI3K/Akt signaling pathway [36]. However, these studies did not address whether Rab11 plays a role in insulin-independent transport of Glut-4 in cardiomyocytes. In addition, we observed that knock-down of Tsg101 in the myocardium impaired plasma levels of Glut-4 under both basal and ischemic conditions. Given that: (1) Tsg101 is a key component of ESCRT-I (endosomal sorting complex required for transport I) [37], and (2) disrupting the function of ESCRT-III (i.e., Vps4 and CHMP3) in rat adipocytes leads to reduced Glut-4 translocation to the plasma membrane in response to insulin [38], these previous findings, together with the present work, suggest that the ESCRT machinery may positively regulate Glut-4 translocation to the cell surface in either insulin-dependent or insulin-independent manners.

With respect to increased total and membrane levels of Glut-4 in Tsg101-TG hearts, it may be explained by the interaction of Tsg101 with Glut-4, at least in part, leading to increased trafficking of Glut-4 to recycling endosomes rather than to lysosomes for degradation. Indeed, we recently showed that Tsg101 could bind to FIP3 (Rab11-family interacting protein 3) and recruit IGF-1R for the endosomal-recycling processes, as FIP3 is an endosomal recycling compartment (ERC) protein that is critical for the structural integrity of the ERC and actively involves the endosomal-recycling [24]. Similarly, Ismaili et al. reported that Tsg101 could interact with and stabilize gluco-corticoid receptor (GR) in HeLa cells, resulting in higher levels of GR at the cell membrane when Tsg101 is over-expressed [39]. Likewise, Kantamneni et al. observed that Tsg101 binding to GISP (G protein-coupled receptor interacting scaffold protein) increased its partner GABA_B_ receptor stability in HEK293 cells [40]. On the other hand, it is also plausible that Tsg101 may be involved in the ubiquitination of Glut-4, as Tsg101 possesses an ubiquitin (Ub) E2 variant (UEV) domain that can bind Ub [37]. Furthermore, ischemic/hypoxic conditions may enhance Glut-4 ubiquitination in cardiomyocytes, which in turn would increase its association with Tsg101 and as a result, may increase its total levels and its translocation to the plasma membrane. More studies would be needed to explore such possibilities in the future.

There are several limitations to this study. First, considering that sarcolemma Glut-4 was up-regulated in Tsg101-TG hearts (Figure 2D,E) and Ad.Tsg101-NRCMs (Figure 4A,B), and was down-regulated in Tsg101-KD hearts (Figure 5D,E) and Ad.shTsg101-cells (Figure 6A,B), it was very surprising for us to observe that glucose uptake was unchanged in either Tsg101-overexpressing or Tsg101-deficient cardiomyocytes relative to their respective controls under basal conditions. This might be explained by our experimental protocol, as we performed our in vitro and ex vivo glucose uptake assays in non-insulin stimulated conditions. Specifically, ex vivo hearts were devoid of circulatory insulin that may induce glucose uptake in non-ischemic conditions. In vitro, cells were starved for 4 h prior to the glucose uptake assay. Hence, control cells were not stimulated with insulin or hypoxic conditions. In addition, the myocardium utilizes fatty acid rather than glucose as its major energy substrate under basal conditions. Therefore, under pre-I/R state, the discrepancy in glucose uptake between Tsg101-TG/KD and their respective control hearts would be too small to show a difference. However, ischemic hearts switch their substrates from fatty acid to glucose for ATP generation. This may explain why glucose uptake was unchanged in Tsg101-TG/KD hearts under normal physiological conditions but was remarkably altered upon ischemia. While ischemic hearts are insulin-resistant and rely on endosomal recycling of Glut-4, a more in-depth investigation is still needed to ascertain the role of Tsg101 in Glut-4 translocation in the presence or absence of insulin stimulation. Second, our recent work reported that Tsg101 protein levels were downregulated in I/R hearts after 45-min ex vivo no-flow ischemia and 1-h reperfusion [25]. In the present study, we observed that Tsg101 levels were upregulated after 45-min ischemia, suggesting an initial increase of Tsg101 protein levels in the ischemic phase and a decrease in the reperfusion phase. The reduction of Tsg101 at the end of the 1-h reperfusion phase may contribute to the detrimental effects and cardiac tissue damage observed in the ex vivo I/R hearts. Hence, overexpression of Tsg101 in mouse hearts and cardiomyocytes may alleviate the deleterious effects during ischemia and hypoxia, thereby rendering protection from tissue damage and cardiomyocyte death after reperfusion. In the same light, we cannot exclude the effects of Tsg101 on other signaling pathways (i.e., Nrf2, Parkin-mediated mitophagy) [25,26] that may have contribution to the protective effects of Tsg101 in this study. Finally, we cannot exclude the contributions of AMPK-dependent pathway to the increased membrane levels of Glut-4 in the ischemic myocardium, as ischemic conditions can activate AMPK-mediated Glut-4 translocation to the sarcolemma [41,42]. Furthermore, Glut-1 has been shown to recycle through endosomal compartments [43] and its distribution may also be altered in cardiomyocytes when Tsg101 expression is elevated or reduced. However, using immunogold labeling method, Davey et al. observed that Glut-1 was predominantly localized in the capillary endothelial cells, whereas Glut-4 was found predominantly in cardiac myocytes [44]. Therefore, the present study only focused on testing whether Tsg101 affected Glut-4 sorting and re-distribution in the heart.

## 5. Conclusions

In summary, we have demonstrated that overexpression of cardiac Tsg101 is able to promote sarcolemma accumulation of Glut-4 in cardiomyocytes after ischemic challenge, leading to enhanced glucose uptake, ATP generation and cardiac functional recovery. This study may provide new strategies for the treatment of ischemic heart disease.
